# A study of clinical profile and outcomes of paediatric patients with acute myeloid leukaemia

**DOI:** 10.3332/ecancer.2026.2064

**Published:** 2026-01-20

**Authors:** Suhani Barbhuiyan, Munlima Hazarika, Asif Iqbal, Manasa Kakunje, Helie P Raval, Anupam Sarma, Roopam Deka

**Affiliations:** 1 Department of Medical and Paediatric Oncology, Dr. B. Borooah Cancer Institute, Guwahati 781016, India; 2 Department of Oncopathology, Dr. B. Borooah Cancer Institute, Guwahati 781016, India; 3 Department of Pathology, All India Institute of Medical Sciences (AIIMS), Guwahati 781101, India

**Keywords:** AML, clinical outcome

## Abstract

**Objective:**

To describe the clinical and socio-demographic profile, as well as the induction outcomes, of paediatric patients with acute myeloid leukaemia (AML).

**Methods:**

A retrospective analysis was conducted of patients up to 18 years of age who presented between 1 January 2017 and 31 December 2021. Patients with a diagnosis of de novo AML confirmed by flow cytometry, or with extra-medullary soft tissue masses proven to be AML by immunohistochemistry, were included. All records were obtained from the Paediatric Oncology database and supplemented by treatment files.

**Results:**

Among 56 patients analyzed, the male-to-female ratio was 1.15. The median age was 7 years (range: 0.2–18 years). The white blood cell (WBC) count at presentation ranged from 1,250/μL to 296,000/μL (median: 29,000/μL). Sixty percent of patients were malnourished, and the majority belonged to lower socioeconomic strata. Fever was the most common presenting symptom (93%), followed by bleeding (61%), easy fatigability (53%), bone pain (46%), hepatosplenomegaly (39%) and lymphadenopathy (14%). According to European LeukaemiaNet (ELN) 2017 risk stratification, 52% of patients were categorized as favourable risk, 18% as intermediate risk and 28% as high risk; one patient could not be risk-stratified. Remission was achieved in 47% of patients, 23% did not achieve remission, 16% died during induction and 14% abandoned treatment.

**Conclusion:**

Demographic variables, nutritional status, WBC count and clinical presentations were not associated with induction outcomes, possibly due to the availability of extensive supportive care services such as accommodation near the hospital, free food and treatment and psychosocial support in the paediatric oncology unit. In contrast, ELN risk categories were strongly associated with outcomes.

## Introduction

Acute leukaemia is the most common paediatric cancer and the second leading cause of cancer-related deaths (after brain and nervous system tumours) among children and adolescents younger than 20 years [1]. In India, cancer is the ninth most common cause of death among children aged 5–14 years [2] and approximately 45,000 children are diagnosed with cancer annually [3]. A significant gap exists between the outcomes of acute lymphoblastic leukaemia (ALL) and acute myeloid leukaemia (AML) in India compared with those reported from high-income countries (HICs) [4, 5].

AML is characterized by clonal neoplastic proliferation of myeloid precursor cells in the bone marrow, with arrest of their maturation. Replacement of marrow by leukaemic blasts leads to anemia, thrombocytopenia and granulocytopenia, with or without leukocytosis. This accumulation of non-functional blast cells inhibits normal hematopoiesis, which, if untreated, results in bone marrow failure and death. Although the etiology of AML is unknown in most cases, several risk factors have been implicated, many of which cause DNA damage—consistent with the understanding that acute leukaemia arises from acquired genetic abnormalities in bone marrow cells. Reported risk factors include germline predisposition, radiation exposure, prior exposure to alkylating agents and electromagnetic radiation [6]. Epidemiological studies have suggested associations between childhood AML and factors such as maternal age, birth weight, previous fetal loss, birth order, maternal pesticide exposure and maternal alcohol use during pregnancy [7].

Three major reasons account for poor outcomes in children with acute leukaemias: treatment abandonment, relapse and treatment-related mortality [7, 8]. For AML, no clear biological differences have been demonstrated between patients in India and those in HICs, indicating that optimal treatment and supportive care are key to improving outcomes [9]. There is a paucity of literature from this geographic region [10], and given that AML is an acquired disease, region-specific factors may influence disease presentation, treatment response and toxicity profiles.

### Justification for the study

Despite India’s high paediatric cancer burden, there is limited published data on paediatric AML from the North–East region. Regional factors—including genetic background, nutritional status, socioeconomic constraints and healthcare accessibility—may significantly impact presentation, tolerance to therapy and outcomes. Understanding these patterns is crucial to tailoring supportive care strategies, optimizing treatment protocols for resource-limited settings and guiding policy interventions aimed at reducing mortality in this underrepresented population.

Treatment with cytarabine and an anthracycline remains the backbone of AML therapy. At Dr. B. Borooah Cancer Institute (BBCI), all patients under 18 years are treated in the Paediatric Oncology department and receive a uniform induction regimen of 2–3 days of anthracycline combined with 5–7 days of infusional cytarabine. This uniformity allows observed outcomes to be more reflective of patient- and disease-related factors rather than treatment heterogeneity. This study was undertaken to generate region-specific data on paediatric AML and to serve as a reference for future clinical and epidemiological research.

## Materials and methods

### Primary objectives

1. To describe the socio-demographic and clinical profile of paediatric patients with AML.
2. To determine induction mortality, rates of complete remission and induction failure in these patients.

A retrospective analysis was conducted on patients presenting to the Paediatric Oncology Unit, Department of Medical and Paediatric Oncology, BBCI, Guwahati, between 1 January 2017 and 31 December 2021. Patients aged ≤18 years were included. Eligible patients had a diagnosis of *de novo* AML confirmed by flow cytometry of blood or bone marrow, or an extra-medullary soft tissue mass proven to be AML by immunohistochemistry.

Patients were excluded if they had:

- Acute leukaemia of ambiguous lineage or mixed phenotypic acute leukaemia.
- Secondary AML arising from a previous myeloproliferative disorder (e.g., juvenile myelomonocytic leukemia and chronic myeloid leukemia).
- Acute promyelocytic leukaemia.
- Incomplete treatment or follow-up details.

Demographic, clinical and follow-up data were extracted from the Paediatric Oncology database and supplemented with treatment files. Records with incomplete, ambiguous or missing information were excluded.

Socio-demographic and clinical variables studied included age, gender, ethnicity, state and district of residence, distance from home to BBCI, socioeconomic status (assessed using the Modified B.

G. Prasad Scale, 2019) and nutritional status (assessed using World Health Organisation Z-score growth charts). Presenting symptoms and signs—including fever, bleeding, easy fatigability, bone pain, lymphadenopathy, hepatosplenomegaly and proptosis—were recorded.

Risk stratification was performed according to the European LeukaemiaNet (ELN) 2017 criteria.

### Statistical analysis

Quantitative data were summarized using mean or median values, as appropriate; qualitative data were expressed as percentages. Paired *t*-test and chi-square test were used to assess associations between variables and outcomes. Data were analyzed using R version 4.2.0 software.

### Definitions

- **Induction mortality:** Death due to any cause within 30 days of starting induction chemotherapy.
- **Complete remission:** Restoration of normal hematopoiesis, transfusion independence, absence of circulating blasts and bone marrow blasts/hematogones <5% of all nucleated cells.
- **Failure of induction:** Non-achievement of complete remission (CR) despite two induction chemotherapy cycles.
- **Abandonment:** Refusal to start induction or discontinuation of treatment before bone marrow evaluation for remission.

All definitions were based on morphological examination of the first pull of bone marrow aspirates. Minimal residual disease assessment by flow cytometry was not performed in this study.

### Summary of study design

- **Design:** Retrospective descriptive study.
- **Place of study:** BBCI, Guwahati, Assam, India.
- **Study period:** January 2017–December 2021 (5 years).
- **Study population:** Paediatric patients (≤18 years) with AML.
- **Inclusion criteria:**
  1. Patients ≤18 years with *de novo* AML confirmed by flow cytometry.
  2. Patients ≤18 years with extra-medullary soft tissue mass proven to be AML by immunohistochemistry.
- **Exclusion criteria:**
  1. Patients >18 years.
  2. No documented evidence of AML surface antigen expression.
  3. Acute leukaemia of ambiguous lineage or mixed phenotypic acute leukaemia.
  4. Acute promyelocytic leukaemia.

### Ethical clearance

This study was approved by the Institutional Ethics Committee of BBCI. The requirement for individual informed consent was waived due to the retrospective nature of the study.

## Results

This retrospective audit included paediatric patients diagnosed with AML and treated in the Paediatric Oncology Unit of the Department of Medical and Paediatric Oncology at BBCI, Guwahati. Data from patients registered between **1 January 2017** and **31 December 2020** were analyzed. Only patients with confirmed diagnoses by surface antigen characterization and complete blood investigation and induction treatment details were included. A total of **56 patients** were analyzed.

Tables 1–6 show the socio-demographic profile of the patients in the study

### Clinical parameters relevant to outcomes

The clinical parameters analyzed for their relevance to treatment outcomes included:

White blood cell (WBC) count at diagnosis

Clinical presentation

Biologic risk categories (ELN 2017)

The majority of patients presented with fever (92.8%), bleeding (60.7%) and easy fatigability (53.5%).

Other common findings included:

Bone pain: 46.4%

Hepatosplenomegaly: 39.2%

Lymphadenopathy: 28.5%

Proptosis: 25%.

The WBC count at presentation ranged from 1,250 × 10⁹/L to 296,000 × 10⁹/L (median: 29,000 × 10⁹/L; mean: 58,000 × 10⁹/L). For analysis, patients were grouped as follows:

≤50,000 × 10⁹/L: 64.2%

50,000–100,000 × 10⁹/L: 20%

100,000 × 10⁹/L: 16%.

### Risk stratification (ELN 2017)

Favourable: 29 patients (52.2%)

High risk: 16 patients (28.5%)

Intermediate risk: 10 patients (17.8%)

Not categorized: 1 patient (1.8%)

### Induction outcomes

Of the 56 patients analyzed:

CR: 26 (46.4%)

No remission: 13 (23.2%)

Death during induction: 9 (16.0%)

Treatment abandonment: 8 (14.2%)

### Associations between risk factors and outcomes

#### Risk category versus outcome

A strong statistical association was observed between ELN risk category and induction outcome (Fisher’s *p* = 0.0001; Cramer’s *V* = 0.512).

High-risk patients were more likely to experience non-remission or death.

Favourable-risk patients were more likely to achieve remission.

#### WBC count at presentation versus outcome

No statistically significant association was found (**Fisher’s *p* = 0.568; Cramer’s *V* = 0.163**).

- Higher WBC counts (>100,000 × 10⁹/L) showed a trend toward poorer outcomes, but without statistical significance.

#### WBC count versus risk category

No significant association observed (**Fisher’s *p* = 0.211; Cramer’s *V* = 0.289**).

#### Nutritional status versu outcome

No significant relationship was noted (**Fisher’s *p* = 0.810; Cramer’s *V* = 0.193**).

- Severe malnutrition was present in 23.2% of patients, but this did not significantly influence induction outcome.

#### Socio-economic status versus outcome

No significant association observed (**Fisher’s *p* = 0.701; Cramer’s *V* = 0.212**).

#### Age group versus outcome

No significant association found (**Fisher’s *p* = 0.077; Cramer’s *V* = 0.332**).

- However, a trend toward higher remission rates was observed in patients >10 years.

#### Gender versus outcome

No statistically significant relationship was found (**Fisher’s *p* = 0.853; Cramer’s *V* = 0.084**).

### Summary of associations

- **Only ELN risk category** showed a statistically significant association with induction outcomes.
- Other demographic and clinical factors (WBC count, nutritional status, socio-economic status, age and gender) did not significantly influence induction remission, non-remission or mortality rates in this cohort.

## Discussion

AML in children is a biologically heterogeneous malignancy associated with substantial risk of mortality [1, 5, 20]. Standard therapy consists of intensive induction chemotherapy followed by consolidation or allogeneic hematopoietic stem cell transplantation, while targeted and hypomethylating agents remain largely investigational in paediatric AML [5, 6]. In our cohort, all patients received the standard ‘7 + 3’ regimen—3 days of daunorubicin with 7 days of cytarabine—or acceptable protocol modifications based on clinical status.

Our analysis included 56 paediatric AML patients treated from 2017 to 2021. The male-to-female ratio (1.15:1) and median age of 7 years align with other Indian and international studies [11–19]. Most patients (78%) were from Assam, with the remainder from other Northeast Indian states and Bangladesh, reflecting the center’s role as a regional referral facility. Fourteen ethnic groups were represented, the largest being Bengali (37%) and Assamese (21%), highlighting the genetic diversity of the catchment population.

Leukocyte count at diagnosis is a recognized prognostic marker in AML [20–23]. In our study, median WBC was 29,000/µL, with 64.2% presenting below 50,000/µL. Although hyperleukocytosis has been linked to worse outcomes in other series [22, 23], we did not observe a statistically significant association, similar to the findings of Morais *et al* [24].

Nutritional status is another potential determinant of outcome. The CCG 2961 trial reported inferior survival in underweight and overweight paediatric AML patients due to higher treatment-related mortality [25]. None of our patients were overweight; however, 60% were malnourished (23% severely). Nutritional status did not significantly influence induction outcomes, possibly reflecting the impact of robust supportive measures at our center, including free treatment, nutritional supplementation, social work support and NGO partnerships.

Host factors such as age, sex, ethnicity and socio-economic status have variably been linked to prognosis [20, 26–28]. In our study, none showed a statistically significant association with induction response, consistent with Gupta *et al* [15], but differing from studies that demonstrated age- or sex-related effects[26–28]. This may again be attributable to comprehensive institutional supportive care reducing disparities in treatment tolerance.

The clinical presentation pattern—fever (93%), bleeding (61%), easy fatigability (53%), bone pain (46%), hepatosplenomegaly (39%), lymphadenopathy (14%) and proptosis (25%)—was broadly in line with other paediatric AML cohorts [16, 17, 29, 30]. ELN 2017 risk categorization revealed a predominance of favourable-risk patients (52%), followed by high (28%) and intermediate (18%) risk. This contrasts with previous Indian and international series where intermediate or high-risk categories dominate [14, 29, 30], likely due to selection bias, as very high-risk patients may be referred directly to transplant-capable centers.

Induction outcomes comprised complete remission in 47%, no remission in 23%, induction death in 16% and treatment abandonment in 14%. Literature from both developed and developing countries shows remission rates of 56%–83% and induction mortality between 3% and 25% [16, 17, 29–42]. When patients who abandoned therapy were excluded, only ELN risk category retained a statistically significant association with outcome: high-risk patients were more likely to fail induction or die, while favourable-risk patients achieved higher remission rates. This association has been consistently reported in prior studies [30–33].

In summary, our findings reinforce the prognostic importance of cytogenetic/molecular risk stratification in paediatric AML, whereas demographic and nutritional parameters were not predictive of induction outcomes in this cohort. The presence of strong supportive care infrastructure may attenuate the influence of socio-demographic risk factors. Multicentre studies in varied resource settings are warranted to determine the generalizability of these results.

## Funding

None.

## Ethical approval

Approved by institutional ethics committee- Dr.B.Borooah Cancer Institute.

## Ethical declaration

None.

## Author contributions

Dr.Suhani Barbhuiyan- Concept, Design, Data Collection, Maintaining master file, Drafting final report, Assigning duties to the study team, Communication with IEC.

Dr.Munlima Hazarika-Design, Interpretation of data, Drafting final report, Publication.

Dr.Asif Iqbal- Concept, Interpretation of data, Statistical analysis.

Dr.Manasa Kakunje- Data Collection, Maintaining master file.

Dr. Helie P.Raval- Data Collection, Maintaining master file.

## Data availability

In EMR and Hard copy at room 15, OPD block, BBCI.

## Study registration

None.

## Figures and Tables

**Figure 1.**
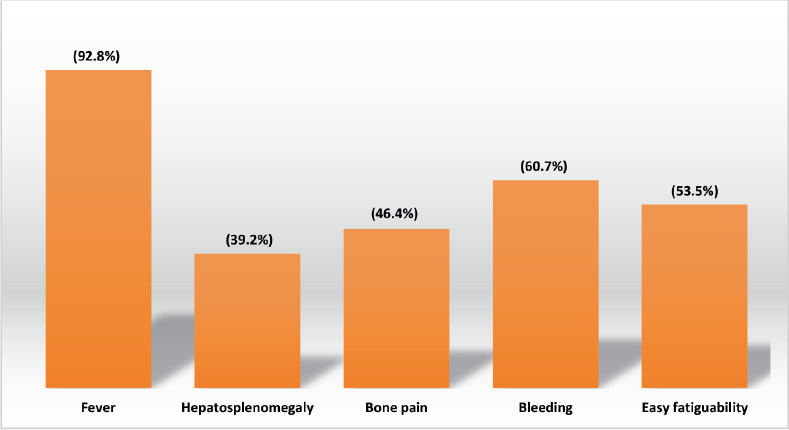
Clinical presentation at diagnosis.

**Figure 2.**
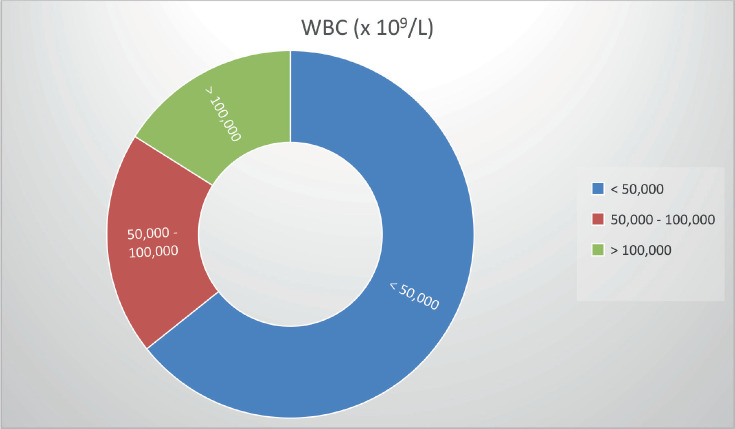
WBC counts at presentation.

**Figure 3.**
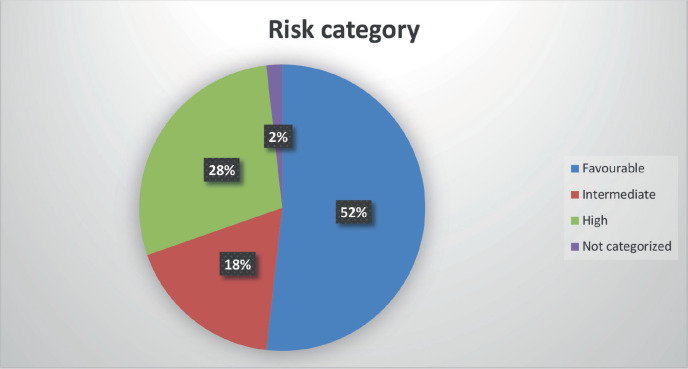
Risk stratification.

**Figure 4.**
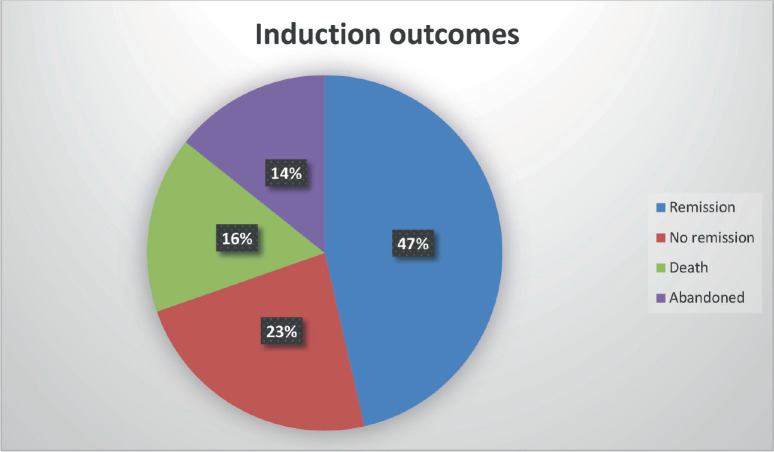
Induction outcomes.

**Table 1.** Socio-demographic profile of patients.

Variable	Min.	Max.	Median	Mean
Age (years)	0.2	18.0	7.00	7.78
Distance from centre (km)	5.0	500.0	240.00	245.35
WBC (×10⁹/L)	1,258	296,260	29,000	58,815.68
Hemoglobin (g/dL)	2.0	12.3	7.65	7.50
Platelets (×10⁹/L)	1,000	340,000	30,000	47,053.5

WBC: white blood cell.

**Table 2.** State of origin.

State	Frequency	Percentage
Arunachal Pradesh	2	3.56%
Assam	44	78.56%
Bangladesh	1	1.78%
Meghalaya	5	8.93%
Nagaland	4	7.14%
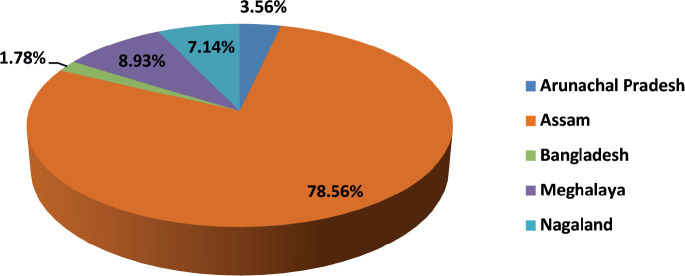		

**Table 3.** Ethnicity.

Ethnicity	Frequency	Percentage
Adi	1	1.78%
Ahom	2	3.57%
Assamese	13	23.21%
Bengali	21	37.49%
Bihari	1	1.78%
Bodo	2	3.56%
Chutiya	1	1.78%
Garo	2	3.56%
Karbi	1	1.78%
Khasi	3	5.36%
Manipuri	1	1.78%
Naga	4	7.14%
Nepali	1	1.78%
Rajbongshi	3	5.36%
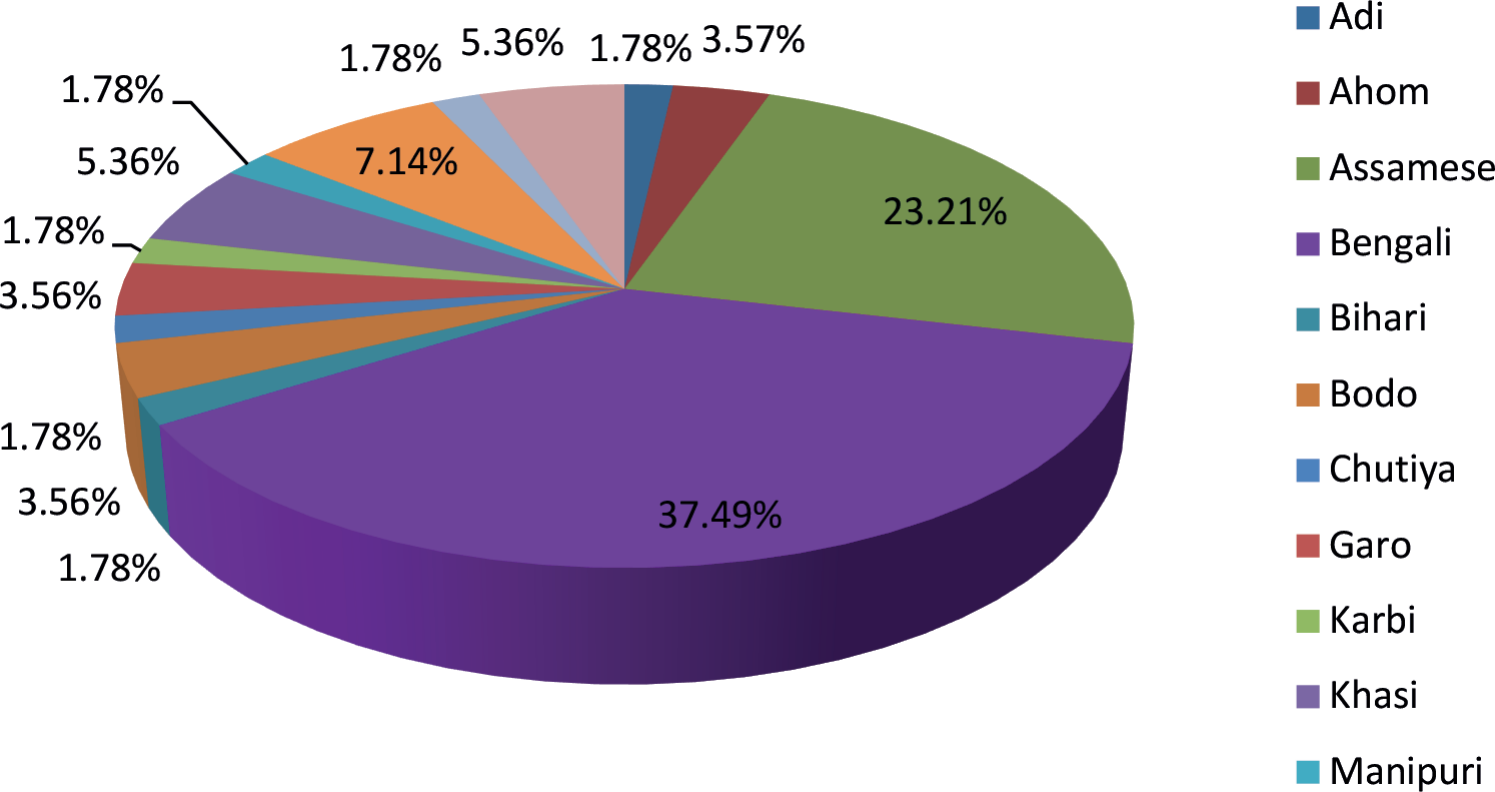		

**Table 4.** Age groups and gender.

Category	Frequency	Percentage
Female	26	46.4%
Male	30	53.5%
≤10 years	39	69.6%
>10 years	17	30.3%
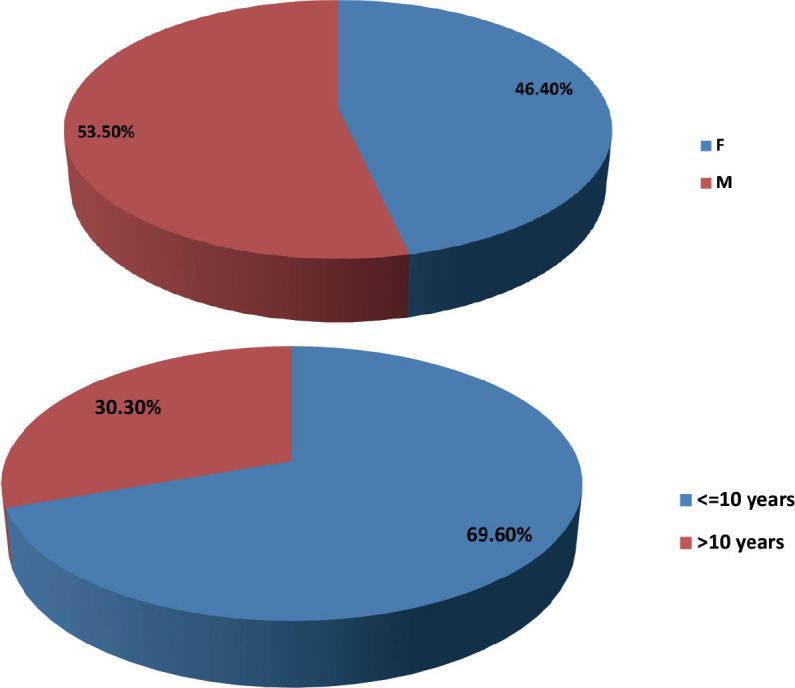		

**Table 5.** Nutritional status.

Nutritional status	Frequency	Percentage
Mild malnutrition	5	8.92%
Moderate malnutrition	16	28.57%
Severe malnutrition	13	23.21%
Well-nourished	22	39.28%
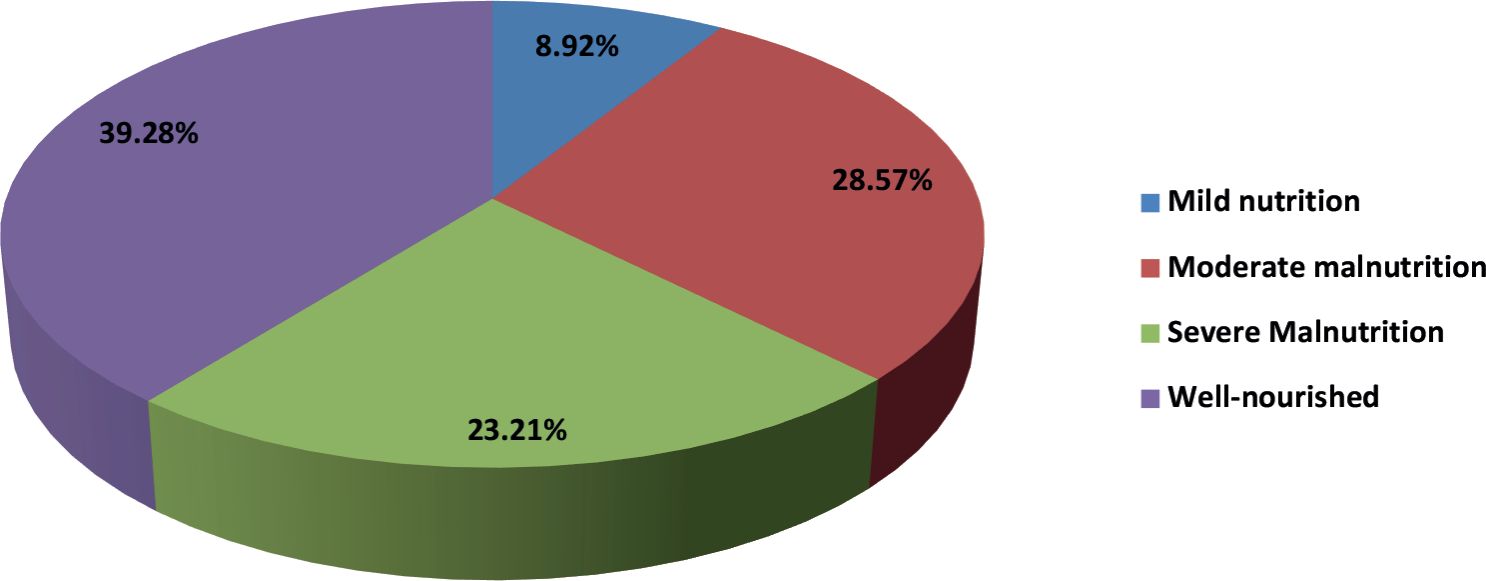		

**Table 6.** Socio-economic status.

Socio-economic class	Frequency	Percentage
Lower	8	14.28%
Lower-middle	31	55.36%
Middle	13	23.21%
Upper-middle	4	7.14%

**Table:** 

Association between risk and outcome				
Risk	Outcome			Total
	Death	No remission	Remission	
Favourable	1 4.3 %	6 26.1 %	16 69.6 %	23 100 %
High	8 53.3 %	5 33.3 %	2 13.3 %	15 100 %
Intermediate	0 0 %	1 11.1 %	8 88.9 %	9 100 %
Not defined	0 0 %	1 100 %	0 0 %	1 100 %
Total	9 18.7 %	13 27.1 %	26 54.2 %	48 100 %
*χ*^2^ = 25.192 · df = 6 · Cramer’s *V* = 0.512 · **Fisher’s *p* = 0.0001**				

Association between WBC and outcome				
WBC- categories	Outcome			Total
	Death	No remission	Remission	
<50,000	5 16.1 %	7 22.6 %	19 61.3 %	31 100 %
50,000–1,00,000	2 20 %	3 30 %	5 50 %	10 100 %
>1,00,000	2 28.6 %	3 42.9 %	2 28.6 %	7 100 %
Total	9 18.8 %	13 27.1 %	26 54.2 %	48 100 %
*χ*^2^ = 2.558 · df = 4 · Cramer’s *V* = 0.163 · Fisher’s *p* = 0.568				

Association between risk and WBC categories					
WBC_categories	Risk				Total
	Favourable	High	Intermediate	Not defined	
<50,000	16 51.6 %	7 22.6 %	7 22.6 %	1 3.2 %	31 100 %
50,000–1,00,000	2 20 %	6 60 %	2 20 %	0 0 %	10 100 %
>1,00,000	5 71.4 %	2 28.6 %	0 0 %	0 0 %	7 100 %
Total	23 47.9 %	15 31.2 %	9 18.8 %	1 2.1 %	48 100 %
*χ*^2^ = 8.041 · df = 6 · Cramer’s *V* = 0.289 · Fisher’s *p* = 0.211					

Association between malnutrition and outcome				
Malnutrition	Outcome			Total
	Death	No remission	Remission	
Mild nutrition	0 0 %	1 25 %	3 75 %	4 100 %
Moderate malnutrition	1 7.7 %	4 30.8 %	8 61.5 %	13 100 %
Severe Malnutrition	3 30 %	3 30 %	4 40 %	10 100 %
Well nourished	5 23.8 %	5 23.8 %	11 52.4 %	21 100 %
Total	9 18.8 %	13 27.1 %	26 54.2 %	48 100 %
*χ*^2^ = 3.579 · df = 6 · Cramer’s *V* = 0.193 · Fisher’s *p* = 0.810				

Association between SE status and outcome				
SE status	Outcome			Total
	Death	No remission	Remission	
L	0 0 %	2 33.3 %	4 66.7 %	6 100 %
LM	6 20.7 %	9 31 %	14 48.3 %	29 100 %
M	3 30 %	1 10 %	6 60 %	10 100 %
UM	0 0 %	1 33.3 %	2 66.7 %	3 100 %
Total	9 18.8 %	13 27.1 %	26 54.2 %	48 100 %
*χ*^2^ = 4.303 · df=6 · Cramer’s *V* = 0.212 · Fisher’s *p* = 0.701				

Association between age groups and outcome				
Age_categories	Outcome			Total
	Death	No Remission	Remission	
≤10 years	5 15.6 %	12 37.5 %	15 46.9 %	32 100 %
>10 years	4 25 %	1 6.2 %	11 68.8 %	16 100 %
Total	9 18.8 %	13 27.1 %	26 54.2 %	48 100 %
*χ*^2^ = 5.288 · df = 2 · Cramer’s *V* = 0.332 · Fisher’s *p* = 0.077				

Association between gender and outcome				
Gender	Outcome			Total
	Death	No remission	Remission	
F	5 20.8 %	7 29.2 %	12 50 %	24 100 %
M	4 16.7 %	6 25 %	14 58.3 %	24 100 %
Total	9 18.8 %	13 27.1 %	26 54.2 %	48 100 %
*χ*^2^ = 0.342 · df = 2 · Cramer’s *V* = 0.084 · Fisher’s *p* = 0.853				
